# The Histopathological Features of the Explanted Lungs from an End-Stage COVID-19 Patient

**DOI:** 10.1080/20961790.2020.1807128

**Published:** 2020-09-01

**Authors:** Kai Li, Youjia Yu, Ding Li, Changxing Luan, Li Hu, Jie Wang, Jingjing Ding, Yanfang Yu, Hang Yang, Jingyu Chen, Feng Chen, Chuan Su

**Affiliations:** Department of Forensic Medicine, Nanjing Medical University, Nanjing, China; Department of Forensic Medicine, Nanjing Medical University, Nanjing, China; Department of Forensic Medicine, Nanjing Medical University, Nanjing, China; Department of Forensic Medicine, Nanjing Medical University, Nanjing, China; Department of Forensic Medicine, Nanjing Medical University, Nanjing, China; Department of Forensic Medicine, Nanjing Medical University, Nanjing, China; Department of Forensic Medicine, Nanjing Medical University, Nanjing, China; Department of Forensic Medicine, Nanjing Medical University, Nanjing, China; Wuxi Lung Transplantation Center, Wuxi People’s Hospital Affiliated with Nanjing Medical University, Wuxi, China; Wuxi Lung Transplantation Center, Wuxi People’s Hospital Affiliated with Nanjing Medical University, Wuxi, China; Department of Forensic Medicine, Nanjing Medical University, Nanjing, China; Key Laboratory of Targeted Intervention of Cardiovascular Disease, Collaborative Innovation Center for Cardiovascular Disease Translational Medicine, Nanjing Medical University, Nanjing, China; Department of Forensic Medicine, Nanjing Medical University, Nanjing, China; Department of Pathogen Biology and Immunology, Jiangsu Key Laboratory of Pathogen Biology, Nanjing Medical University, Nanjing, China

Although the pathological findings of coronavirus disease 2019 (COVID-19) associated with acute respiratory distress syndrome (ARDS) have been reported in previous studies, the data of histopathological features of end-stage COVID-19 lungs are still lacking. We have previously reported the clinical features and managements of three COVID-19 patients with irreversible deterioration of pulmonary function who received lung transplantation (LT) after being supported by extracorporeal membrane oxygenation (ECMO) [1].

The detailed history and treatment of the patient has been reported previously [1]. In brief, the male patient was 73 years of age with history of type 2 diabetes mellitus, diabetic nephropathy, chronic renal failure, coronary atherosclerotic heart disease, atrial fibrillation, chronic bronchitis, emphysema and deafness. He was admitted to the hospital with the initial symptoms of fever and coughs and was diagnosed as COVID-19 by reverse real-time PCR assay on Feb 2, 2020. The patient gradually developed ARDS during hospitalization and received tracheostomies and invasive mechanical ventilation (MV) on Feb 20. Veno-venous (VV)-ECMO was established on Feb 21. Continuous renal replacement therapy (CRRT) started on Feb 26 due to the rapid increase of serum creatine. Echocardiography (Mar 9) revealed right ventricular failure, biatrial enlargement (left atrium diameter, LAD 43 mm), mild mitral insufficiency, mild aortic insufficiency, moderate tricuspid insufficiency, reduced left ventricular diastolic function (mitral valve orifice E peak < A peak), and elevated pulmonary artery pressure (tricuspid annular plane systolic excursion, TAPSE 12 mm; flow velocity integral of E wave, TVIE peak < A peak; pulmonary artery systolic pressure, PASP 53 mmHg; ejection fraction, EF 56.01%). Given that the results of throat swab tests of severe acute respiratory syndrome coronavirus-2 (SARS-CoV-2) had been continuously negative since Feb 25, the patient received LT after establishing intraoperative central cannulated veno-arterial (VA)-ECMO on Mar 10. He was weaned off intraoperative VA-ECMO support after LT. And the pre-LT VV-ECMO was maintained with low-dose inotropes until Mar 12. The patient was extubated on Apr 2.

After fixation by buffered formalin, 42 tissue blocks were sampled from the whole lungs according to the locations and were numbered as shown in Figure 1A. All blocks were embedded in paraffin and cut into slides. Hematoxylin & eosin (HE) staining and Masson’s trichrome staining were performed. The histopathological features of the lungs included diffused pulmonary fibrosis (Figure 1B), alveolar edema and hemorrhage, reactive type II pneumocytes hyperplasia, interstitial thickening, and lymphocytes, monocytes and neutrophils infiltration, indicating ARDS, which were also observed in previous report [2–5]. In most residual alveoli, there were highly swollen macrophages with swallowed lipids in the cytoplasm (Figure 1C). Multinucleated giant cells accumulated in residual airspaces (Figure 1D). Multifocal bronchial epithelial cell hyperplasia accompanied by squamous metaplasia was noted. Large amount of secretary vesicles containing mucin were present within bronchial mucosal surface, suggesting hyperplasia and hypersecretion of goblet cells (Figure 1E). Mixture of mucus, exudates, red blood cells and macrophages and neutrophils blocked in bronchia (Figure 1F). Unlike the other reports, no hyaline membrane formation was observed, indicating the prolonged and progressive inflammatory process.

**Figure 1:**
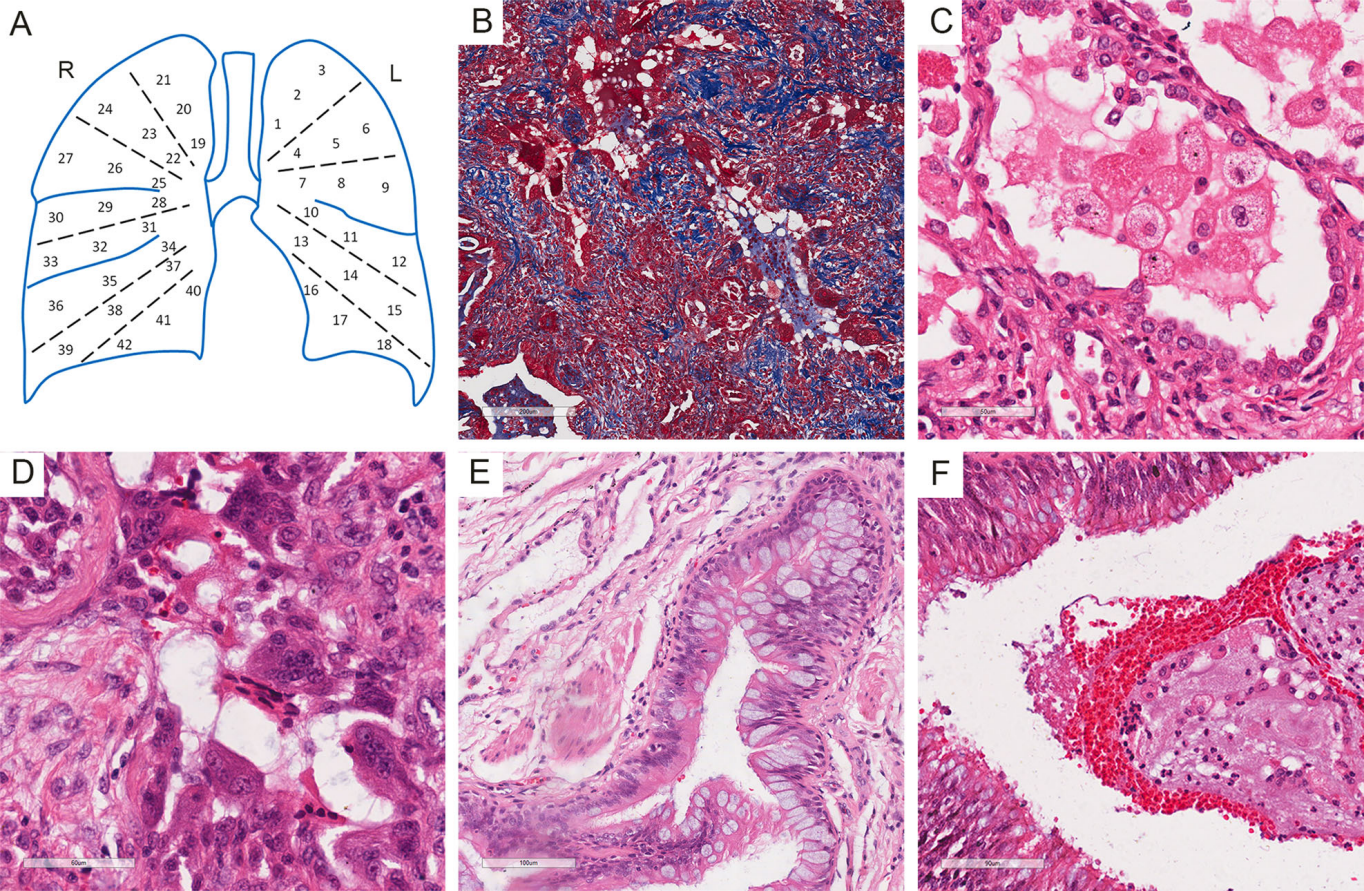
Histopathologic examination of bilateral lungs. (A) Illustration of sampling strategy. (B) Masson’s trichrome staining showed reconstruction of pulmonary tissue and diffused pulmonary fibrosis. (C) Swollen macrophages and reactive type II pneumocyte hyperplasia. (D) Multinucleated giant cells accumulated in residual airspaces. (E) Hyperplasia and hypersecretion of goblet cells. (F) mixture of mucus, exudates, red blood cells, macrophages and neutrophils blocked in bronchia.

Notably, significantly thickened walls and anastomotic stenosis were revealed in arterioles (Figure 2A). Masson’s trichrome staining and immunofluorescence staining with an antibody to α-SMA showed obvious fibrosis and smooth muscle cells hyperplasia in the tunica media layer (Figure 2B, C), indicating severe pulmonary vascular remodeling.

**Figure 2:**
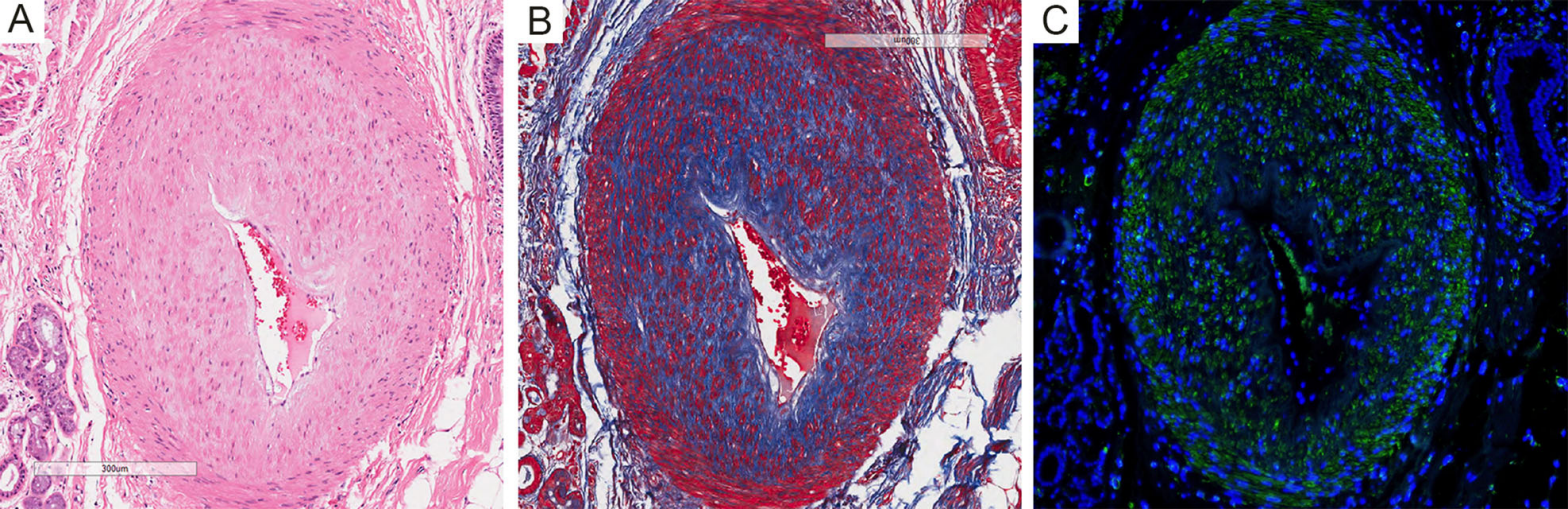
(A) Thickened arteriole wall and anastomotic stenosis. (B) Masson’s trichrome staining showed fibrosis in the tunica media layer. (C) Immunofluorescence staining showed obvious smooth muscle cells hyperplasia. Immunofluorescence staining of smooth muscle cells was performed with a mouse anti-α-SMA antibody (1:1 000, ABM, Y414854). Nuclei were stained by DAPI (Sigma, D9542).

The detailed histopathologic changes of the explanted lungs from an end-stage COVID-19 patient, who was supported by VV-ECMO for 18 days before LT, are consistent with his clinical manifestations. Our findings show more severe consequences of alveolar damage and tissue repair caused by SARS-CoV-2 comparing to previous reports, while the histopathologic changes may also be partially due to the intensive medical supports such as mechanical ventilation and prolonged course of the disease after VV-ECMO establishment. Particularly, the patient developed significant pulmonary vascular remodeling and pulmonary hypertension (PH) during the progression of COVID-19, which could be secondary to inflammatory lesions of the lung parenchyma. Considering the general conditions of the patient, the advanced age, underlying pulmonary and heart diseases might contribute to PH pathogenesis.

Our findings provide comprehensive histopathological features of end-stage COVID-19 and pathologic evidence of secondary PH as a complication of COVID-19.

## References

[1] Chen JY Qiao K Liu F , et al. Lung transplantation as therapeutic option in acute respiratory distress syndrome for COVID-19-related pulmonary fibrosis. Chin Med J (Engl). 2020;133:1390–1396. 32251003 10.1097/CM9.0000000000000839 PMC7339336

[2] Zhang Y Gao Y Qiao L , et al. Inflammatory response cells during acute respiratory distress syndrome in patients with Coronavirus Disease 2019 (COVID-19). Ann Intern Med. 2020;L20–0227. 10.7326/L20-0227 PMC7175423 32282871

[3] Tian S Hu W Niu L , et al. Pulmonary pathology of early-phase 2019 novel coronavirus (COVID-19) pneumonia in two patients with lung cancer. J Thorac Oncol. 2020;15:700–704. 32114094 10.1016/j.jtho.2020.02.010 PMC7128866

[4] Xu Z Shi L Wang Y , et al. Pathological findings of COVID-19 associated with acute respiratory distress syndrome. Lancet Respir Med. 2020;8:420–422. 32085846 10.1016/S2213-2600(20)30076-X PMC7164771

[5] Barton LM Duval EJ Stroberg E , et al. COVID-19 Autopsies, Oklahoma, USA. Am J Clin Pathol. 2020;153:725–733. 32275742 10.1093/ajcp/aqaa062 PMC7184436

